# Effectiveness of single loading dose of dexmedetomidine combined with propofol for deep sedation of endoscopic retrograde cholangiopancreatography (ERCP) in elderly patients: a prospective randomized study

**DOI:** 10.1186/s12871-022-01630-8

**Published:** 2022-03-28

**Authors:** Mo Chen, Yi Sun, Xueyan Li, Chun Zhang, Xiaochen Huang, Yiming Xu, Chengyong Gu

**Affiliations:** Department of Anesthesiology, Gusu School, the Affiliated Suzhou Hospital of Nanjing Medical University, Suzhou Municipal Hospital, Nanjing Medical University, No.242 Guangji Road, Suzhou, Jiangsu China

**Keywords:** Dexmedetomidine, Propofol, Deep sedation, Endoscopic retrograde cholangiopancreatography, Elderly patients

## Abstract

**Background:**

Endoscopic retrograde cholangiopancreatography (ERCP) is an advanced endoscopic procedure and requires deep sedation. Deep sedation with dexmedetomidine for the respiratory drive preserved has become popular in recent years. However, the use of dexmedetomidine in elderly patients is controversial because its adverse events are more common. The objective of this study was to investigate the effectiveness of a single loading dose of dexmedetomidine combined with propofol for deep sedation of ERCP in elderly patients.

**Methods:**

In this prospective randomized trial, 49 elderly patients undergoing ERCP were randomly allocated to the dexmedetomidine (DEX) or propofol (PRO) groups. The single loading dose of dexmedetomidine was set at 0.5 μg/kg at the start of anesthesia induction and loading for 10 min. The primary outcome was the cumulative dose of propofol. Secondary outcomes included time to awake, the frequency of airway interventions, and hemodynamics.

**Results:**

The intraoperative cumulative dose of propofol was lower in the DEX group (111.0 ± 12.6 μg/kg/min) than the PRO group (143.7 ± 23.4 μg/kg/min) (*P* < 0.001). There was no statistically significant difference in the time to awake between the two groups. The incidence of artificial airway interventions and hypotension in the PRO group (36%, 60%) were significantly higher than those in the DEX group (4.2%, 16.7%) (*P* = 0.011, *P* = 0.003, respectively). In addition, the occurrence of bradycardia increased significantly in the DEX group (58.3%) compared with the PRO group (12%) (*P* < 0.001).

**Conclusions:**

The single loading dose of dexmedetomidine combined with propofol can reduce propofol consumption and artificial airway intervention and provide better hemodynamic stability than propofol for deep sedation in elderly patients during ERCP.

**Trial registration:**

www.chictr.org.cn (Registration number ChiCTR1900028069, Registration date 10/12/2019).

## Background

Endoscopic retrograde cholangiopancreatography (ERCP) is an advanced endoscopic procedure that allows diagnostic and therapeutic purposes in biliary and pancreatic disease, such as gallstone extraction and stent placement [1]. To patients, the ERCP is an uncomfortable and painful procedure and requires deeper sedation and fewer body movements than routine gastrointestinal endoscopy. The combination of propofol and an opioid analgesic is the preferred sedative solution over the last decade [2]. However, increased propofol in deep sedation usually accompanies incremental cardiopulmonary complications, such as respiratory depression, hypoxemia, and hypotension, causing more perioperative concerns when anesthetizing elderly patients in a prone position [3, 4].

Dexmedetomidine is a highly selective α_2_- adrenoceptor agonist and is utilized widely for sedative and anxiolytic properties by decreasing the activity of noradrenergic neurons. Unlike propofol, dexmedetomidine provides sedation with the respiratory drive preserved even at a loading dose [5]. Many studies have evaluated the effect of dexmedetomidine and propofol on sedation for gastrointestinal endoscopic procedures [6, 7]. Mukhopadhyay et al. found dexmedetomidine could reduce propofol requirement and provide a more stable level of sedation during ERCP procedure [8]. However, it was proved that dexmedetomidine infusion alone was not as effective as propofol on sedation quality and was associated with significantly low blood pressure and heart rate and prolonged recovery time [9, 10]. These prominent adverse events are increasingly raising concern about the use of dexmedetomidine in elderly patients who need better stable hemodynamics. The effectiveness and safety of dexmedetomidine with propofol during ERCP require further studies to adequately evaluated in elderly patients.

Considering the biphasic effect of dexmedetomidine on blood pressure with lower readings at lower concentrations and higher readings at higher concentrations due to peripheral α_2_-receptor stimulation, we speculated that the low blood pressure and heart rate might be associated with continuous intravenous infusion during ERCP procedure, not the injection of loading dose [11]. This study prospectively evaluated the effectiveness and safety of a single loading dose of dexmedetomidine combined with propofol during ERCP in elderly patients.

## Methods

### Patients

This prospective single-blinded randomized clinical trial was approved by the Institutional Ethics Committee of Suzhou Municipal Hospital (KL901071) and has been registered in the Chinese Clinical Trial Registry (Registration No. ChiCTR1900028069, Registration date 10/12/2019). Written informed consent was obtained from each patient or legal surrogate before inclusion. This study was conducted from July 2020 to March 2021, in accordance with the Helsinki Declaration of the World Medical Association.

After informed consent, patients who planned for ERCP aged over 65 years were evaluated for eligibility with an American Society of Anesthesiologists (ASA) physical status of I-III. Exclusion criteria were known allergy to any drug used in this study, heart rate of fewer than 50 beats/min, treated with beta-blockers, uncorrected shock, oxygen saturation measured by pulse oximetry (SpO_2_) less than 90%, left ventricular ejection fraction of less than 50%, kidney or hepatic insufficiency.

### Randomization and sedation procedure

Patients were randomized to the propofol (PRO) group or the dexmedetomidine (DEX) group in a 1:1 ratio by using the PLAN procedure of the SAS 9.4 software (SAS Institute, Cary, USA). The allocation information was sealed in sequentially numbered opaque envelopes. Although the anesthesiologists who collected the data during ERCP were not blinded, they were not involved in follow-up outcome analysis.

All patients were prone to continuous oxygen (2 L/min) and were treated by one experienced endoscopist. During ERCP, the SpO_2_, heart rate, and electrocardiogram were continuously monitored, the noninvasive blood pressure was measured every 3 min.

According to previously published trials, all patients received sufentanil (0.1 μg/kg) and propofol (1 to 2 mg/kg) for anesthesia induction based on ideal body weight [6]. In the DEX group, the intravenous loading dose of dexmedetomidine (Yangtze River, China) was set at 0.5 μg/kg at the start of anesthesia induction and loading for 10 min. In both groups, anesthesia was maintained with continuous infusion of propofol (target plasma concentration 2 to 4 μg/ml) (Diprifusor, AstraZeneca, UK). The intraoperative plasma concentration of propofol was left to the anesthesiologist responsible for the patient. The depth of anesthesia was continuously monitored, and maintained a bispectral index between 40 and 60 (Conview, Pearlcare, China). The infusion of propofol was stopped at the end of ERCP.

Artificial airway interventions were performed when respiratory depression (SpO_2_ < 90%) occurred. If there was no improvement after chin lift/jaw thrust manipulation, nasal airway and endotracheal intubation were sequentially applied. Bradycardia was defined as heart rate was 50 beats/min or less. Hypertension was defined as mean arterial pressure (MAP) higher than 110 mmHg or a 20% increase from the baseline. Hypotension was defined as MAP lower than 65 mmHg or 20% less than the baseline. In both groups, catecholamine was administered when hypotension occurred, and atropine was administered when the heart rate was 50 beats/min or less.

### Outcome

The primary outcome of this study was to compare the cumulative dose of propofol in the two groups during ERCP. The secondary outcomes included the following: time to awake, the frequency of airway interventions (chin lift/jaw thrust, nasal airway, endotracheal intubation), the frequency of administration of catecholamine and atropine, and the frequency of occurrence of hypertension, arrhythmia, and cardiac arrest.

### Statistical analysis

The sample size calculation was based on the primary outcome of the cumulative dose of propofol during ERCP. According to a previous study about the reduced consumption of propofol by dexmedetomidine during ERCP [12], we calculated the sample size and found 26 patients each group were required considering a 10% dropout rate (power = 0.8, α = 0.05).

Statistical analyses were carried out using the SPSS 20.0 statistical software (IBM Corporation, Armonk, NY, USA). Quantitative data were expressed in the mean ± standard deviation and analyzed with the Student’s *t*-test or Welch’s *t*-test. Categorical data were expressed as a frequency and percentage and analyzed with the Fisher’s exact test or the Chi-square test. A *P*-value of less than 0.05 was considered statistically significant.

## Results

**Fig. 1:**
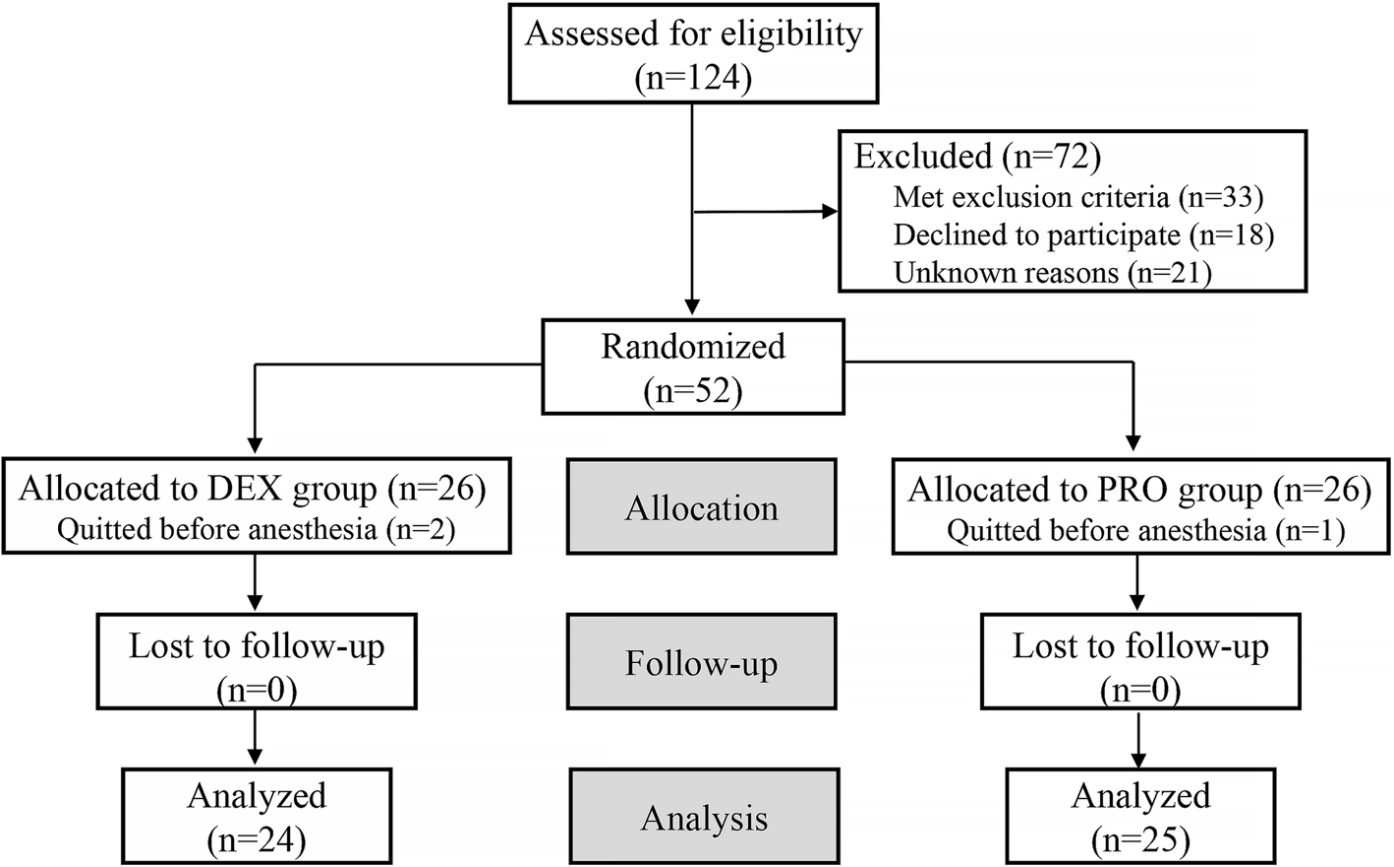
Consort diagram

The consort diagram of this study was shown in Fig. 1. Of 124 patients assessed for eligibility, 72 patients were excluded for the exclusion criteria and declined to participate. Eventually, 49 patients in the two groups were analyzed.

There were no statistically significant differences between the PRO and DEX groups regarding age, gender, BMI, ASA physical status, heart rate, and blood pressure (Table 1).

**Table 1:** Characteristics of the patients at baseline

	PRO group (*n* = 25)	DEX group (*n* = 24)	*P*-value
Age (y)	73.0 ± 5.9	72.0 ± 6.1	0.532
Gender (M/F)	14/11	14/10	0.869
BMI (kg/m^2^)	23.5 ± 3.0	23.7 ± 2.9	0.843
ASA I/II/III, n	0/17/8	0/17/7	0.830
Heart rate (beats/min)	75.8 ± 8.5	73.0 ± 8.0	0.249
Blood pressure (mmHg)			
Systolic	138.4 ± 16.5	137.4 ± 13.3	0.805
Diastolic	79.0 ± 9.4	76.7 ± 9.8	0.844
Mean	98.8 ± 10.2	96.9 ± 10.2	0.976

Data are presented as mean ± standard deviation or number

*BMI* Body mass index, *ASA* American society of anesthesiologists

Intraoperative data were summarized in Table 2. The ERCP time, anesthesia time, and time to awake were similar between the PRO and DEX groups. However, the intraoperative cumulative dose of propofol was lower in the DEX group than in the PRO group (*P* < 0.001).

**Table 2:** Intraoperative data

	PRO group (*n* = 25)	DEX group (*n* = 24)	*P*-value
ERCP time (min)	47.4 ± 18.1	49.0 ± 18.2	0.747
Anesthesia time (min)	52.1 ± 17.9	53.9 ± 18.3	0.724
Time to awake (min)	11.3 ± 1.7	11.9 ± 1.6	0.225
Cumulative dose of propofol (μg/kg/min)	143.7 ± 23.4	111.0 ± 12.6	< 0.001

Data are presented as mean ± standard deviation

*ERCP* Endoscopic retrograde cholangiopancreatography

**Fig. 2:**
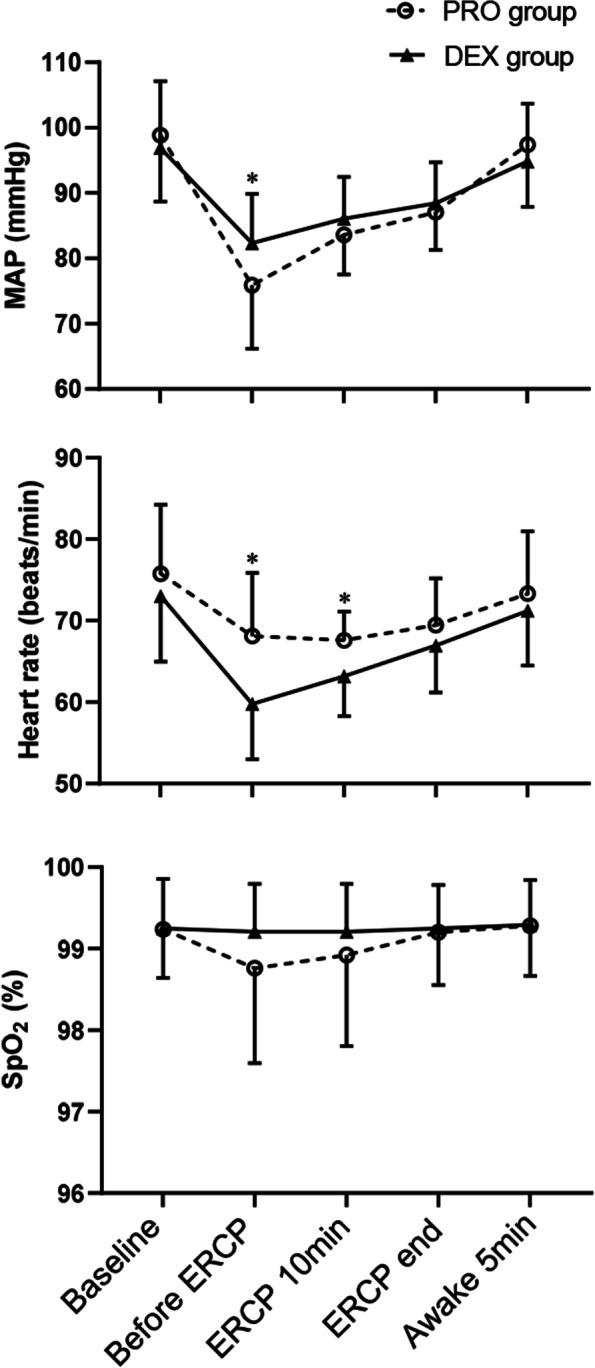
Mean arterial pressure, heart rate, and SpO_2_ at different time points during ERCP. Data are presented as mean ± standard deviation. *MAP* Mean arterial pressure

As shown in Table 3, the incidence of artificial airway interventions and hypotension in the PRO group were significantly higher than those in the DEX group (*P* = 0.011, *P* = 0.003, respectively). In addition, the occurrence of bradycardia increased significantly in the DEX group compared with the PRO group (*P* < 0.001). No arrhythmia and cardiac arrest were observed in the two groups. The MAP in the PRO group was lower than that in the DEX group before ERCP, while the heart rate in the PRO group was higher before ERCP and 10 min after ERCP (^*^P < 0.05) (Fig. 2).

**Table 3:** Comparison of the adverse events

	PRO group (*n* = 25)	DEX group (*n* = 24)	*P*-value
Artificial airway interventions	9 (36)	1 (4.2)	0.011
Chin lift/jaw thrust	8	1	
Nasal airway	1	0	
Endotracheal intubation			
Hypotension (vasopressor)^*^	15 (60)	4 (16.7)	0.003
Bradycardia (atropine)^*^	3 (12)	14 (58.3)	< 0.001
Hypertension	4 (16)	5 (20.8)	0.725
Arrhythmia	0 (0)	0 (0)	
Cardiac arrest	0 (0)	0 (0)	

Data are presented as number (%)

^*^Data are presented as the number of episodes with vasopressor or atropine administration

## Discussion

Our results suggest that the combined use of a single loading dose of dexmedetomidine could better preserve the respiratory drive and hemodynamic stability than propofol in elderly patients during ERCP. In this study, the single loading dose of dexmedetomidine reduced propofol consumption and the need for artificial airway intervention over propofol. Furthermore, the time to awake has not been significantly prolonged in elderly patients. Our study recommended using a single loading dose of dexmedetomidine combined with propofol in elderly patients for deep sedation of ERCP.

The ERCP is a very uncomfortable and painful procedure with significant stimulation and usually needs deep sedation. In recent years, the combination of propofol and an opioid analgesic became the standard for deep sedation during ERCP, with the advantage of rapid induction, shorter time to awake, better tolerance, and satisfaction even in elderly patients [2, 13]. However, the side effects of propofol, such as airway obstruction, hypoxia, and hypotension, are typical for the narrow therapeutic window [3, 14–16]. Elderly patients are more sensitive to propofol and its respiratory adverse effects and dose-dependent hypotension [17–19]. The increased incidence of upper airway obstruction becomes more troublesome and may be challenging due to shared airways for ERCP procedures [3]. Therefore, we chose to use dexmedetomidine in elderly patients for its sedation with respiratory drive preservation.

Dexmedetomidine is a highly selective α2- adrenoceptor agonist. Its distribution half-life is approximately 6 min, and it has an elimination half-life of 2 h [20, 21]. Previous studies compared the use of dexmedetomidine with propofol for deep sedation during ERCP. Dexmedetomidine can also be used alone or combined with other drugs. However, dexmedetomidine alone was insufficient as propofol and not recommended [22, 23]. Therefore, we chose to combine dexmedetomidine with propofol. Similar to our results, in a prospective randomized study, the addition of dexmedetomidine reduced propofol requirement, provided a better stable level of sedation, and increased anesthetist satisfaction [8]. Abdalla et al. also reported that dexmedetomidine-propofol combination during ERCP showed better hemodynamic stability [24]. Taken together, these results suggest that the use of dexmedetomidine resulted in lesser propofol consumption, better efficacy, and stable sedation. Our study further evaluated the efficacy and safety of dexmedetomidine in different types of patients as elderly patients.

In our study, we administered a single loading dose of dexmedetomidine for 10 min to reduce the propofol consumption. Even though a recent study concluded that dexmedetomidine and propofol sedation produce similar upper airway collapsibility, our results found less artificial airway intervention even at a loading dose of dexmedetomidine as previous studies [5, 6, 25]. We speculate that the reason may be the decreased propofol consumption during anesthesia induction. Unlike our and other research results [9, 26], in a previous study, Muller et al. showed a more significant reduction in blood pressure in patients undergoing ERCP and concluded dexmedetomidine was associated with greater hemodynamic instability [27]. Based on the biphasic effect of dexmedetomidine on blood pressure, dexmedetomidine was used only as a single loading dose to counteract the decreased blood pressure produced by propofol during anesthesia induction in our study [11, 28]. To overcome hypotension, a frequent side effect of dexmedetomidine, we did not infuse the dexmedetomidine continuously at a low rate during ERCP. One of our concerns about using dexmedetomidine in elderly patients is delayed time to awake. Though Muller et al. showed the dexmedetomidine was associated with a prolonged recovery period, in our study, we did not observe significant differences between the two groups in the time to awake [27]. This result may be due to not continuous infusion of dexmedetomidine and reduced propofol consumption. In this study, bradycardia is also a frequent side effect of dexmedetomidine and was usually observed during anesthesia induction.

There are several limitations in our study. First, this is a single-center study of a relatively small size. Second, we didn’t early detect hypoxemia and apnea. Capnography can provide an early warning sign by measuring end-tidal carbon dioxide in patients under deep sedation and should be used in further studies. Third, sufentanil, a prescription opioid, could induce respiratory depression, especially with propofol. More suitable analgesics with less respiratory depression used in elderly patients during ERCP deserve further research.

## Conclusions

The single loading dose of dexmedetomidine combined with propofol reduces propofol consumption, and artificial airway intervention provides better hemodynamic stability than propofol for deep sedation in elderly patients during ERCP. Moreover, the time to awake has not been significantly prolonged by the single loading dose of dexmedetomidine.

## Data Availability

All relevant data used and/or analyzed during the current study are available from the corresponding author on reasonable request.

## Abbreviations

ASA: American society of anesthesiologists
ERCP: Endoscopic retrograde cholangiopancreatography
SpO_2_: Oxygen saturation measured by pulse oximetry
BMI: Body mass index
MAP: Mean arterial pressure
